# What rheumatologists in the United States think of complementary and alternative medicine: results of a national survey

**DOI:** 10.1186/1472-6882-10-5

**Published:** 2010-01-28

**Authors:** Nisha J Manek, Cynthia S Crowson, Abigale L Ottenberg, Farr A Curlin, Ted J Kaptchuk, Jon C Tilburt

**Affiliations:** 1 Division of Rheumatology, Department of Medicine, Mayo Clinic, 200 First St., Rochester, MN 55905, USA; 2 Division of Biomedical Statistics and Informatics, Mayo Clinic, 200 First St., Rochester, MN 55905, USA; 3 Bioethics Research Program. Mayo Clinic, 200 First St., Rochester, MN 55905, USA; 4 Section of Internal Medicine and the MacLean Center for Clinical Medical Ethics, The University of Chicago, 5758 S. Maryland Ave, Chicago, IL 60637, USA; 5 Osher Research Center, Harvard Medical School, 401 Park Dr., Boston, MA 02215, USA; 6 Division of General Internal Medicine, Department of Medicine, Mayo Clinic, 200 First St., Rochester, MN 55905, USA

## Abstract

**Background:**

We aimed to describe prevailing attitudes and practices of rheumatologists in the United States toward complementary and alternative medicine (CAM) treatments. We wanted to determine whether rheumatologists' perceptions of the efficacy of CAM therapies and their willingness to recommend them relate to their demographic characteristics, geographic location, or clinical practices.

**Methods:**

A National Institutes of Health-sponsored cross-sectional survey of internists and rheumatologists was conducted regarding CAM for treatment of chronic back pain or joint pain. In this study we analyzed responses only from rheumatologists. Response items included participant characteristics and experience with 6 common CAM categories, as defined by the National Institutes of Health. Descriptive statistics were used to describe attitudes to CAM overall and to each CAM category. Composite responses were devised for respondents designating 4 or more of the 6 CAM therapies as "very" or "moderately" beneficial or "very likely" or "somewhat likely" to recommend.

**Results:**

Of 600 rheumatologists who were sent the questionnaire, 345 responded (58%); 80 (23%) were women. Body work had the highest perceived benefit, with 70% of respondents indicating benefit. Acupuncture was perceived as beneficial by 54%. Most were willing to recommend most forms of CAM. Women had significantly higher composite benefit and recommend responses than men. Rheumatologists not born in North America were more likely to perceive benefit of select CAM therapies.

**Conclusions:**

In this national survey of rheumatologists practicing in the United States, we found widespread favorable opinion toward many, but not all, types of CAM. Further research is required to determine to what extent CAM can or should be integrated into the practice of rheumatology in the United States.

## Background

Results of the National Health Interview Surveys in 2002 and 2007 showed that complementary and alternative medicine (CAM) use among patients with arthritis is very pervasive [1,2]. The odds ratio for ever-use of CAM among adults with arthritis was 1.59 compared with adults with no chronic diseases and was higher than CAM use among patients with any other common chronic diseases including cardiovascular disease and cancer [3]. It is clear, therefore, that patients' use of CAM is an important clinical issue; this is especially true for rheumatologists and primary care physicians who treat arthritis.

The relationship of physicians to CAM has been the subject of some international study. A meta-analysis by Ernst et al in 1995 [4] and a review by Astin et al in 1998 [5] have summarized this research. The proportion of mainstream physicians who incorporated some form of CAM into their own practices (drawn from 19 methodologically acceptable studies) ranged from 9% to 19%, and CAM referral rates ranged from 4% to 43% [5]. More recent surveys from countries such as Italy and Germany indicate an increasing trend of primary physicians incorporating CAM into their practices [6,7].

In the past 5 years, the conventional medical community has shown increasing openness toward several CAM therapies. For example, much-awaited research on popular nutraceuticals, specifically on the use of glucosamine and chondroitin sulfate for knee osteoarthritis [8], has been published in prominent medical journals, but few positive results have been shown. These trends in increasing awareness of CAM in general have not been without controversy and highlight broader debates about what constitutes the scope of acceptable contemporary medical practice including rheumatology. These and other issues may affect whether rheumatologists consider referring their patients for CAM therapies or even using CAM in their clinical practice.

Rheumatology is often algorithm and protocol driven, and adherence to guidelines and strict standards of safety and efficacy is a professional expectation [9]. However, especially in an era of fewer safe therapeutic prescription options (eg, withdrawal of rofecoxib from the market in 2004), it is interesting to examine to what extent rheumatology specialists consider less-rigorously tested CAM modalities to be legitimate therapeutic elements in clinical practice.

We are aware of 1 published survey of rheumatologists in the United States that examined the extent to which physicians incorporated 22 CAM therapies into their professional practices [10]. This survey, undertaken in 2000, included questions about dietary prescriptions and exercise intervention, which are considered in the realm of conventional medical care [11].

In the current study, we surveyed rheumatologists in the United States and addressed several broad research questions. 1) What are the prevailing attitudes and practices of conventional rheumatologists regarding common types of CAM? 2) How do rheumatologists' perceptions of CAM efficacy and their willingness to recommend CAM relate to their demographics, geographic location, and clinical practices? 3) What variables predict rheumatologists' attitudes toward CAM?

## Methods

### Participants

As of 2005 and 2006, the total number of practicing rheumatologists in the United States was estimated to be 4,946 [12]. In the summer of 2007, a 12-page self-administered questionnaire was mailed to a stratified random sample of 600 rheumatologists younger than 65 years in the United States as part of a larger national survey of health care providers' perceptions about CAM, which was sponsored by the National Institutes of Health. Details of the survey have been published elsewhere [13]. Participants were screened to determine if they were currently in practice. Using the standard conservative survey research definitions, response rates were calculated (RRI from the American Association for Public Opinion Research) [14]. This survey study was evaluated and declared exempt by the Mayo Clinic Institutional Review Board.

### Survey Instrument

The survey was developed through a formal process which included focus groups, drafting of an instrument, cognitive testing, and further revision of the instrument, as described in detail previously [13]. The survey focused on the rheumatologists' opinions and use of 6 common CAM therapies: spinal manipulation (eg, chiropractic care), acupuncture, energy medicine (eg, Reiki), meditation practice (eg, yoga), glucosamine and/or chondroitin, and body work (eg, massage, shiatsu). These 6 CAM groups were drawn from existing National Institutes of Health categories of CAM and from testing of physicians' degree of familiarity during focus groups.

### Independent Variables

Respondents reported their demographic information including age, sex, region of birth (North America, Central/South America/Caribbean, Europe/Australia, Middle East, Africa, Asia/Pacific Islands), race, rheumatology practice characteristics (solo, private; group, private; institutional, private; academic), and region of practice in the United States.

### Dependent Variables

We measured 2 dimensions of physicians' attitudes toward integrating CAM into their clinical practice. These were 1) perceived benefit and 2) likelihood of recommending each of the 6 CAM therapies for treatment of chronic back pain or joint pain. Perceived benefit of each of the CAM therapies was surveyed with the questionnaire item: "How beneficial do you think each of these therapies is for chronic back pain or joint pain?" A 4-point scale with response categories "very," "moderately," "not very," or "not at all" beneficial was used.

Similarly, likelihood of recommending each CAM therapy was surveyed with the questionnaire item: "How likely would you be to recommend each of these therapies as one component of a comprehensive treatment package for patients with chronic back pain or joint pain?" A 4-point scale with response categories "very likely to," "somewhat likely to," "not very likely to," and "would never" recommend was used.

### Data Management and Analysis

Descriptive statistics were used to summarize the data. Cronbach's α was used to measure how well the variables of perceived benefit of and likelihood of recommending CAM modalities were interrelated.

Proportional odds models (an extension of logistic regression allowing more than 2 ordered categories for the outcome) for each of the 6 CAM modalities using a 0 to 3-point scale were used to examine how rheumatologists' perceptions of the benefit of CAM and their willingness to recommend CAM relate to their demographic, geographic, and clinical practice characteristics. The proportional odds assumption was examined using the score test and was not violated.

A composite response variable was used to analyze which characteristics of rheumatologists are independently associated with their attitudes toward CAM overall by using responses to items for individual types of CAM. In this case, perceived benefit was defined as respondents indicating 4 or more of the 6 CAM therapies as being either "very beneficial" or "moderately beneficial." Similarly, a composite response was defined for the likelihood of recommending CAM, defined as respondents indicating that they were either "very likely" or "somewhat likely" to recommend 4 or more of the 6 CAM therapies. Multivariate logistic regression models were used to examine the association between these composite responses and the independent variables of interest. For all tests of association, a 2-tailed alpha, *P *< .05 was considered significant.

## Results

Of the 600 rheumatologists in the United States randomly sampled, 345 (58%) responded to the survey. The mean age of the respondents was 52 years (range, 25-65 years), 80 (23%) were women, and the majority (89%) were white (Table 1). This demographic distribution of the respondents was comparable to the national estimates of rheumatologists in 2005, with a median age of 51 years and 30% female practitioners [12]. Most of the rheumatologists worked in a group practice setting (46%), followed by solo practice (28%), academic (20%), and institutional (3%) settings. A plurality of respondents (140, 41%) practiced in the Northeast region of the United States (Table 1). The majority (82%) of the rheumatologists responding were born in North America. Respondents and nonrespondents did not differ by age, race, sex, practice setting, or geographic location.

**Table 1:** Characteristics of 345 Rheumatologists Responding

**Characteristic**^**a**^	**Value**^**b**^
Mean (range) age, y	52 (28-65)
Female sex	80 (23)
Race (n = 333)	
Asian	30 (9)
Black	3 (1)
White	296 (89)
Other	4 (1)
Practice Setting (n = 342)	
Solo	97 (28)
Group	157 (46)
Institutional	11 (3)
Academic	68 (20)
Other	9 (3)
Region	
Northeast	140 (41)
South	136 (39)
Midwest	41 (12)
West	28 (8)
Place of Birth (n = 332)	
North America	272 (82)
Other	60 (18)

^a ^No. of respondents answering the question is indicated if less than 345.

^b ^No. of respondents (%) unless otherwise indicated.

**Figure 1:**
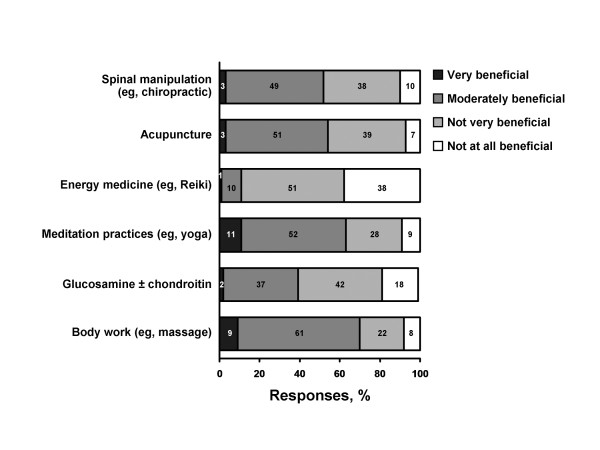
**Survey responses of rheumatologists**. Views of respondents regarding the benefit of various types of complementary and alternative medicine.

Rheumatologists' responses to perceived benefit of each CAM therapy are shown in Figure 1. The CAM modality with the highest perceived benefit was body work (practices such as massage), with 70% of respondents indicating that this was either "very beneficial" or "moderately beneficial"; the modality with the next highest perceived benefit was meditation at 63%. More than half of all respondents perceived acupuncture (54%) and spinal manipulation (52%) to be either "very" or "moderately" beneficial. Glucosamine and/or chondroitin was perceived as beneficial by less than half of the rheumatologists, with more than half (60%) indicating that this was either "not very" or "not at all" beneficial.

**Figure 2:**
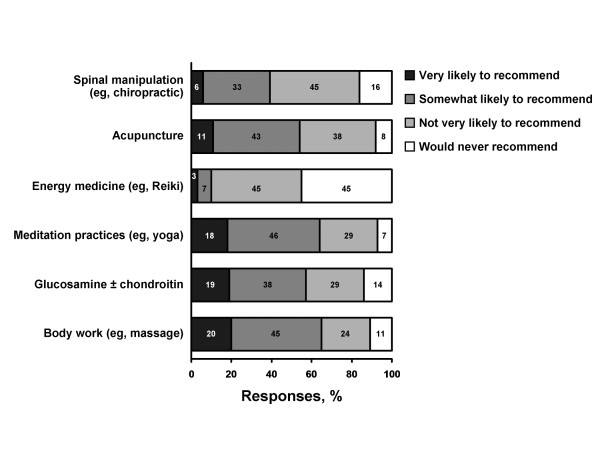
**Survey responses of rheumatologists**. Views of respondents regarding their willingness to recommend various types of complementary and alternative medicine.

Rheumatologists were "very" or "somewhat likely" to recommend body work (65%), followed closely by meditation (64%) (Figure 2). More than half of all respondents were likely to recommend glucosamine and/or chondroitin (57%) and acupuncture (54%). Only 10% of rheumatologists would consider recommending an energy medicine modality (such as Reiki).

The responses for the 6 CAM modalities regarding perceived benefit were highly correlated (Cronbach's α = 0.76), as were the responses for the 6 CAM modalities regarding recommendation for therapy (Cronbach's α = 0.78).

Multivariate analysis for each category of CAM therapy is shown in Table 2. Rheumatologists in institutional practice settings were more likely to perceive benefit of spinal manipulation and body work compared with their counterparts in other practice settings. However, the number of respondents in institutional practice was small (n = 11), and the result was not significant when the categories "other" and "institutional practice" were combined. Women rheumatologists were significantly more likely than men to perceive the benefit of meditation practices (*P *< .001), glucosamine and/or chondroitin (*P *= .003), and body work (*P *= .01). Also, rheumatologists practicing in the South (*P *= .05) and those not born in North America (*P *= .04) were more likely to perceive the benefit of meditation practices.

**Table 2:** Associations Between Characteristics of Rheumatologists and Responses Regarding Perceived Benefit of Each CAM Therapy

	**OR (95% CI)**^**a**^					
	
Characteristic	Spinal Manipulation	Acupuncture	Energy Medicine	Meditation	G ± C	Body Work
Age (per 10 y)	0.8 (0.6-1.1)	0.8 (0.6-1.1)	0.8 (0.6-1.2)	0.8 (0.6-1.1)	1.0 (0.8-1.3)	0.9 (0.6-1.2)
Sex (Female)	1.5 (0.9-2.6)	1.4 (0.8-2.4)	2.0 (0.98-4.0)	**2.6** **(1.5-4.6)**	**2.3** **(1.3-3.9)**	**2.2** **(1.2-4.0)**
Race (White)	1.1 (0.5-2.7)	0.8 (0.3-1.9)	1.4 (0.5-4.3)	1.7 (0.7-4.2)	0.7 (0.3-1.5)	1.4 (0.5-3.5)
Practice Setting (vs Solo)	*P *= .45	*P *= .66	*P *= .87	*P *= .34	*P *= .25	*P *= .30
Group	1.4 (0.8-2.4)	0.9 (0.5-1.6)	1.0 (0.6-2.0)	0.8 (0.5-1.4)	1.3 (0.8-2.2)	1.2 (0.7-2.1)
Institutional/Other	1.0 (0.4-2.6)	0.6 (0.2-1.7)	1.1 (0.3-3.6)	1.2 (0.4-3.4)	1.0 (0.4-2.7)	2.4 (0.8-7.3)
Academic	0.9 (0.5-1.8)	1.2 (0.6-2.3)	0.8 (0.4-1.7)	0.6 (0.3-1.1)	1.9 (1.0-3.6)	0.8 (0.4-1.7)
Region (vs Northeast)	*P *= .98	*P *= .60	*P *= .59	***P *= .05**	*P *= .63	*P *= .29
South	1.0 (0.6-1.6)	0.9 (0.6-1.5)	0.9 (0.5-1.7)	1.7 (1.01-2.7)	1.7 (1.02-2.7)	1.9 (1.1-3.2)
Midwest	0.9 (0.4-1.8)	0.7 (0.3-1.5)	0.6 (0.3-1.4)	0.7 (0.3-1.5)	1.7 (0.8-3.5)	1.2 (0.6-2.6)
West	0.6 (0.3-1.4)	0.6 (0.3-1.5)	0.9 (0.3-2.3)	0.8 (0.4-1.9)	1.1 (0.5-2.5)	1.0 (0.4-2.6)
Place of Birth (not North America)	1.0 (0.5-2.2)	1.2 (0.6-2.5)	1.5 (0.6-3.7)	**2.2** **(1.02-4.5)**	1.0 (0.5-2.1)	1.8 (0.8-4.0)

Abbreviations: C, chondroitin; CAM, complementary and alternative medicine; CI, confidence interval; G, glucosamine; OR, odds ratio.

^a ^OR for an ordinal response of "very," "moderately," "not very," or "not at all" beneficial. Bolded entries indicate statistical significance.

Composite analyses of individual CAM modality ratings showed that 124 of 326 rheumatologists (38%) viewed CAM overall as beneficial (defined as rating 4 or more of the 6 CAM modalities as "very beneficial" or "moderately beneficial"), and 132 of 330 rheumatologists (40%) were willing to recommend CAM therapies (defined as rating 4 or more of the 6 CAM modalities as "very likely" or "somewhat likely" to recommend).

Sex was significantly associated with higher composite "benefit" response (*P *= .02) (Table 3). Women rheumatologists were more likely to rate CAM therapies as beneficial than were men. Rheumatologists not born in North America were more likely to recommend CAM therapies (*P *= .008). No significant differences in benefit or recommend responses were seen for practice setting or geographic region of the United States. Although the association did not reach statistical significance, rheumatologists in the South were slightly more likely to recommend CAM therapies (*P *= .07).

**Table 3:** Multivariate Associations Between Characteristics of Rheumatologists and Responses Regarding Perceived Benefit and Likelihood of Recommending CAM

	OR (95% CI)	
	
Characteristic	**Benefit**^**a**^	**Recommend**^**b**^
Age (per 10 y)	0.9 (0.7-1.3)	0.8 (0.6-1.1)
Sex (Female)	**1.9** **(1.1-3.4)**	1.7 (0.9-2.9)
Race (White)	1.3 (0.5-3.2)	1.5 (0.6-4.0)
Practice Setting (vs Solo)	*P *= .65	*P *= .67
Group	0.9 (0.5-1.6)	1.3 (0.7-2.3)
Institutional/Other	1.3 (0.5-3.8)	0.8 (0.3-2.6)
Academic	0.7 (0.3-1.4)	1.0 (0.5-1.9)
Region (vs Northeast)	*P *= .49	*P *= .07
South	1.3 (0.8-2.2)	1.6 (0.9-2.7)
Midwest	1.5 (0.7-3.4)	0.8 (0.3-1.8)
West	0.8 (0.3-2.1)	0.6 (0.2-1.7)
Place of Birth (not North America)	1.8 (0.8-4.0)	**2.5** **(1.1-5.6)**

Abbreviations: CAM, complementary and alternative medicine; CI,

confidence interval; OR, odds ratio.

^a ^OR for a composite response of 4 or more of 6 CAM therapies rated as "very" or "moderately" beneficial vs 3 or fewer of 6 CAM therapies with the same responses. Bolded entries indicate statistical significance.

^b ^OR for a composite response of 4 or more of 6 CAM therapies rated as "very likely" or "somewhat likely" to recommend vs 3 or fewer of 6 CAM therapies with the same responses. Bolded entries indicate statistical significance.

## Discussion

Results of this national survey showed that most rheumatologists express favorable attitudes toward most categories of CAM practices relevant to the care of patients with chronic back pain or joint pain. More than half of the respondents consider common individual CAM therapies to be beneficial and are at least moderately likely to recommend them. However, rheumatologists' opinions regarding perceived benefit of common CAM therapies and their likelihood of recommending them varied widely across different CAM modalities; percentage of favorable responses ranged from as high as 70% for body work to as low as 11% for energy medicine. After controlling for other factors, female sex and being born outside the United States were independently associated with rheumatologists' favorable ratings of perceived benefit and willingness to recommend CAM.

Because chronic musculoskeletal conditions are a leading indication for the use of CAM, rheumatologists have been urged to discuss CAM with their patients [15]. Provider attitudes seem to be becoming more favorable; a recent study suggests that if rheumatologists use more participatory styles of decision making with patients, patients are more likely to tell them about their CAM use [16]. The main result of our study contributes to the literature from the providers' perspective in that the historical antagonism between CAM practitioners and mainstream rheumatology physicians seems weakened.

We found that female rheumatologists were twice as likely to perceive benefit of common CAM therapies as their male counterparts, a result that is consistent with some but not all previous surveys of other physician groups [17-20]. Sex has been of no clear significance in other studies [21-23]. Although we had no particular a priori hypothesis about a sex difference, it is possible that women, who are more favorably disposed to CAM in general, may carry over that attitude into their professional attitudes [24]. Furthermore, these data showing a sex difference at least suggest that core physician characteristics are integral to their attitudes and clinical reasoning about CAM that cannot be ignored.

Other factors associated with favorable attitudes toward CAM are not well explained from this exploratory study. Although their numbers were small, rheumatologists practicing in an institutional practice were more likely to perceive benefit from spinal manipulation and body work compared with their colleagues in group and academic settings. The reasons for this are not clear from our data. Large institutional practices may incorporate certain CAM treatments into their programs based on the availability of certified practitioners in their area or may have arrangements for reimbursement for particular CAM therapies, thus making it easier for physicians to access and gain a level of familiarity with these practices. It is quite likely that consumer demand for CAM is motivating more insurers and hospitals to incorporate CAM [25-27], which may differentially affect institutional practices.

Results from this national survey did not show regional variations in attitudes toward CAM treatments according to geographic region of the United States. This is consistent with a study by Borkan et al [21], which did not identify a significant difference in belief of effectiveness of CAM among practice locations in the United States. Further corroboration is provided by another study showing that a physician's country of origin did not have a significant effect on his or her belief in CAM [23]. We did, however, find that rheumatologists born outside the United States had more favorable attitudes toward CAM overall. This may reflect ethnic familiarity with some CAM therapies such as meditation.

We found a gradient of acceptance of CAM. Only 11% of respondents considered energy medicine beneficial, and physicians were less likely to recommend treatments such as Reiki. This may reflect to some extent the availability of, or experience with, energy medicine. Also, research in the area of energy medicine is lacking compared with other CAM therapies such as chiropractic, acupuncture, and body work, which may equally influence the lack of legitimacy ascribed to this category of CAM treatment. It is also possible that physicians have difficulty believing in a therapy that they view as scientifically implausible. It is worth noting that the CAM modalities that were most favored are those that appear most regularly in the popular media, which, to some extent, may influence physician choices [28,29].

This study raises larger questions that remain unanswered. For instance, should CAM be "integrated" into the routine treatment options of rheumatologists? If an integrated approach is to be developed that allows a combination of the best of conventional medicine and CAM to provide an informed choice for patients with osteoarthritis, then it must be research led and evidence based. Signs show that CAM is becoming increasingly integrated. The number of randomized trials of CAM treatments is increasing, and the Cochrane Library now includes more than 200 reviews of complementary medicine interventions.

One consequence of the increase in the availability of high-quality data is that guidelines and consensus statements published by conventional medical bodies have supported the value of CAM. For example, current Osteoarthritis Research Society International guidelines list acupuncture and glucosamine with or without chondroitin as nonpharmacologic treatment for hip and knee osteoarthritis [9]. Therefore, it appears that one stimulus for increasing integration has been the increase in research evidence. Nevertheless, many unanswered questions remain before a truly integrated practice of rheumatology would be practically possible, including potential attitudinal trends among rheumatologists themselves.

Notable strengths of this study include a random (representative) sampling of rheumatology providers from defined areas of the United States. This provides a comprehensive view of rheumatology specialist attitudes, whereas previous surveys have mostly focused on primary care providers [6,7,17,19,22,23]. The reliability of the questions in this survey has been rigorously tested. Aspects not previously studied that can influence physician perceptions of CAM, such as ethnic background, practice setting, and geographical region, were examined. Previous physician surveys have tended to define CAM in various ways, from as few as 3 modalities to as many as 25. The definition of CAM in this survey was based on well-described categories by the National Institutes of Health, thus enhancing interpretation of data.

Some limitations of this survey include the quantitative nature of data gathering. A close-ended survey style does not allow for description of the "art of medicine" and decision making. A qualitative study would allow rheumatologists the opportunity to describe and discuss the manner in which they manage common musculoskeletal conditions on a day-to-day basis [30]. This study was cross-sectional, and as CAM therapies evolve and more studies are published, the trend of physician attitudes will likely change. It is important to note that our results may not be generalizable to physicians on the West Coast because the sample of respondents was small compared with that from the Northeast. Additionally, we do not know how physician attitudes may shift and change when treating particular rheumatologic conditions other than osteoarthritis. The response rate of 58% could potentially exaggerate response bias, but responders and nonresponders did not differ significantly. We included only common CAM therapies, potentially underestimating the prevalence of true CAM usage. The incorporation of questions on placebo prescribing could potentially affect the attitudes of the physician toward CAM. Other important considerations that potentially affect rheumatologists' attitudes toward CAM in formulating a treatment recommendation, including patient preferences, clinical experience, and published research, will be addressed in a subsequent analysis.

## Conclusions

The results of this exploratory survey suggest that there is widespread favorable opinion toward many, but not all, types of CAM therapies. The degree to which rheumatologists are likely to consider CAM in formulating a treatment recommendation relates to different aspects of their background, especially sex, country of birth, and characteristics of their practice (eg, institutional practice setting). If CAM therapies are to be more fully integrated into the practice of rheumatology, we need a more extensive assessment of how the modes of reasoning and therapeutic modalities of many CAM traditions can fit in with the diagnostic and therapeutic categories of contemporary rheumatology practice.

## Abbreviations

CAM: complementary and alternative medicine.

## Competing interests

The authors declare that they have no competing interests.

## Authors' contributions

N.J.M. carried out preparation of the manuscript; C.S.C. performed the statistical analysis; A.L.O. helped in the draft of the manuscript. F.A.C. participated in design of the survey and helped draft the manuscript. T.J.K. participated in design of the survey and helped to draft the manuscript. J.C.T. participated in design of the study and helped to draft the manuscript. All authors read and approved the final manuscript.

## Pre-publication history

The pre-publication history for this paper can be accessed here:

http://www.biomedcentral.com/1472-6882/10/5/prepub
